# Centrality and compatibility of institutional logics when introducing value-based reimbursement

**DOI:** 10.1108/JHOM-01-2021-0010

**Published:** 2021-09-15

**Authors:** Thérèse Eriksson, Lars-Åke Levin, Ann-Charlotte Nedlund

**Affiliations:** Department of Health, Medicine and Caring Sciences, Linköping University , Linköping, Sweden

**Keywords:** Value-based reimbursement, Financial incentives, Holistic healthcare, Neo-institutional theory, Institutional logics, External demands, Patient choice

## Abstract

**Purpose:**

Using financial incentives has been criticised for putting too much focus on things that can be measured. Value-based reimbursement may better align professional values with financial incentives. However, professional values may differ between actor groups. In this article, the authors identify institutional logics within healthcare-providing organisations. Further, the authors analyse how the centrality and compatibility of the identified logics affect the institutionalisation of external demands.

**Design/methodology/approach:**

41 semi-structured interviews were conducted with representatives from healthcare providers within spine surgery in Sweden, where a value-based reimbursement programme was introduced. Data were analysed using thematic content analysis with an abductive approach, and a conceptual framework based on neo-institutional theory.

**Findings:**

After the introduction of the value-based reimbursement programme, the centrality and compatibility of the institutional logics within healthcare-providing organisations changed. The logic of spine surgeons was dominating whereas physiotherapists struggled to motivate a higher cost for high quality physiotherapy. The institutional logic of nurses was aligned with spine surgeons, however as a peripheral logic facilitating spine surgery. To attain holistic and interdisciplinary healthcare, dominating institutional logics within healthcare-providing organisations need to allow peripheral institutional logics to attain a higher centrality for higher compatibility. Thus, allowing other occupations to take responsibility for quality and attain the feeling of professional pride.

**Originality/value:**

Interviewing spine surgeons, physiotherapists, nurses, managers and administrators allows us to deepen the understanding of micro-level behaviour as a reaction (or lack thereof) to macro-level decisions.

## Introduction

This article focuses on how institutions within healthcare-providing organisations respond to external demands. These demands can arise as an effect of governance. Governance within healthcare has been strongly influenced by corporate strategies for the last 30 years. Reimbursement programmes serve as a popular tool to affect behaviour in healthcare through financial incentives ( Dranove and White, 1987 ; Conrad, 2015 ; Roland and Campbell, 2014 ; Sharan *et al.* , 2016 ; Kazberouk *et al.* , 2016 ). However, the use of financial incentives has been criticised for putting focus on measurable outcomes and statistics, rather than values that better reflect the quality of care. In order to promote quality-enhancing activities, the use of value-based reimbursement programmes (VBRP) has increased in an attempt to better align financial incentives with professional values. The design of a VBRP should facilitate integrated healthcare to promote a holistic healthcare perspective ( Conrad, 2015 ; Porter, 2010 ).

The decision to implement a value-based reimbursement programme can be considered as a macro-level reform. It has been argued that macro-level reforms require the vigorous support of micro-level leaders if change is to be realised ( Kellogg, 2011 ). As a bridge between these levels of healthcare systems, managers play a crucial role by motivating staff, and give stability through differing strategies ( Oliver, 1991 ; Burnett *et al.* , 2016 ; Korlén *et al.* , 2017 ). However, managers can also function as a buffering zone and dampen the impact of external demands, such as financial incentives in a reimbursement programme ( Ellegard and Glenngard, 2019 ). Previous studies have shown that managerial stability and competence in aligning external demands with an internal strategy, strongly influence healthcare providers' response to financial and quality challenges ( Oliver, 1991 ; Burnett *et al.* , 2016 ). It has been demonstrated that multiple institutional logics exist within organisations ( Dunn and Jones, 2010 ), but little is known about how the relationship between these logics affect the impact of external demands within the healthcare context.

We use the case of a Swedish regional authority where a value-based reimbursement programme (VBRP) was introduced within elective spine surgery. This paper draws on neo-institutional theory and the concept of three institutional pillars ( Scott, 2014 ) in relation to occupation and profession. The introduction of a VBRP does not happen in a vacuum. Hence, organisations cannot be fully understood in isolation from the external influences arising from a wider organisational context as well as political shifts in regulation and governance ( Scott, 2014 ). Thus, institutional theory provides a suitable framework for examining the nature of external demands and the institutional logics present within organisations.

The aim of this paper is to identify institutional logics within healthcare-providing organisations. Further, we aim to analyse how the centrality and compatibility of the identified logics affect institutionalisation of external demands.

## The case of the value-based reimbursement programme

The case is based on the introduction of a value-based reimbursement programme within elective spine surgery in Region Stockholm in 2013. Region Stockholm is the largest of 21 self-governed regions within the Swedish healthcare system. The regions' main responsibility is to provide and finance healthcare, mainly through tax revenues, to provide universal coverage. Both public and private healthcare providers are approved, but to receive public funding, private healthcare providers must establish a commissioning contract with the region in which they wish to deliver care. This is done either through the Public Procurement Act ( SFS, 2016:1145 ) or through the Act on Systems of Choice ( SFS, 2008:962 ) (known as Patient Choice within healthcare). Under the Public Procurement Act healthcare providers have a time-limited authorisation for a certain volume at a negotiated price, unique to each healthcare provider. Whereas Patient Choice is a continuous contract with no restriction on volume but with a set price equal to all providers, making them compete based on quality and ultimately the patients' choice.

Region Stockholm introduced Patient Choice, with a value-based reimbursement programme (VBRP) within elective spine surgery, in 2013. Elective surgery is scheduled in advance and does not involve an emergency. Only private healthcare providers were accredited within VBRP and in 2017 there were four accredited healthcare providers. Two providers were located in Stockholm city, one in a Stockholm suburb and the fourth in a neighbouring region. Despite one of the healthcare providers being geographically located in a different region it did not affect their commissioning contract, all healthcare providers were providing care under the same regulative conditions.

The new commissioning contract was designed in line with value-based health care ( Porter, 2008 ) with a holistic perspective of healthcare, in order to promote integrated care and interdisciplinary assessment of patients. When the surgical procedure is registered, the healthcare provider receives a **prospective payment,** which includes a bundled payment and an expected performance-based payment. The prospective payment is adjusted for patient characteristics to avoid cherry-picking. The **bundled payment** should cover all healthcare utilisation related to the spine surgery (e.g. potential complications, reoperation, rehabilitation), for the full care episode of one year. The idea of bundled payment is to stimulate an effective and integrated care chain by using a fixed payment to the provider for all services provided during the care episode. The **performance-based payment** is based on the outcome measure, Global Assessment (GA), administered by the national quality register Swespine ( Swedish society of spinal surgeons, 2018 ). The measure is a retrospective transition question asked one year after surgery (“How is your back/leg pain today compared to before the surgery?”) ( Parai *et al.* , 2018 ). The patient can choose between six response options (pain free, much better, somewhat better, unchanged, worse, did not have pain before the surgery). More details about the reimbursement programme can be found in a previously published study ( Eriksson *et al.* , 2020 ).

## Theoretical framework

In order to understand how different institutional logics within an organisation affect the institutionalisation of external demands, we use an approach to new-institutional theory based on Scott's conceptual framework ( Scott, 2014 ). This framework describes institutions through three pillars: the regulative, the normative, and the culture-cognitive pillar. The regulative pillar refers to the practice of rule-setting, monitoring, sanctioning and incentivising. It comprises formal legislation but also less formal rule making. Thus, the instrumentalism is emphasised within the regulative pillar. The normative pillar encompasses values and norms. Values are described as “conceptions of the preferred or the desirable”, whereas norms are defined as the scripts for how to reach the desirable goals and what means are legitimate in attaining them. Hence, appropriateness is emphasised within the normative pillar. Lastly, the cultural-cognitive pillar refers to the processes and frameworks of the shared perception, which enable sense making for the professionals when meeting the “external world of stimuli”. Thus, the conformity to established and accepted beliefs (orthodoxy) is emphasised within the culture-cognitive pillar.

If the regulative, normative and culture-cognitive pillars are aligned, they reinforce each other and make the institution more robust. If the pillars are misaligned, they support differing behaviours which can cause an institutional change ( Scott, 2014 ). Misalignment of pillars can arise as an institution faces new demands. Organisations act within an institutional environment that contains several demands ( Thornton *et al.* , 2012 ). The demands could be external, such as a third party payer changing the terms of receiving reimbursement. But demands could also be internal such as healthcare professionals within an organisation changing their attitude on reasonable means to attain their goal. How an organisation is affected by these demands depends on the stabilising and meaning-making properties of the regulative, normative, and cultural-cognitive pillars ( Scott, 2014 ), but also on how multiple institutional logics manifest within an organisation ( Besharov and Smith, 2014 ).

Different institutional logics can exist within a healthcare organisation and be more or less compatible ( Greenwood *et al.* , 2011 ; Dunn and Jones, 2010 ). Institutional logics often overlap, such that actors confront and draw on multiple logics ( Thornton *et al.* , 2012 ). There is a variety of potential areas of disagreement within the healthcare organisation, e.g. over goals, means, appropriate material resources, appropriate human resources, the control of work, or the definition of organisational boundaries ( Scott, 2014 ). Potentially contradictory institutional logics can cause conflicts, e.g. professional logics versus market logics. Many organisations that were traditionally operating in professional partnership mode have been invaded by corporate practices and subjected to greater market controls ( Scott, 2014 ).

Institutional logics manifest differently within organisations. In some organisations multiple institutional logics influence the core mission and strategy of the organisation ( Pache and Santos, 2013 ), whereas other organisations are dominated by a single logic with additional logics being more peripheral ( Jones *et al.* , 2012 ). Some researchers mean that multiple logics in organisations can be harmful and evoke contestation and conflict ( Battilana and Dorado, 2010 ; Zilber, 2002 ), threaten the performance of the organisation and ultimately lead to a collapse ( Tracey *et al.* , 2011 ). Other researchers mean that logics can coexist ( McPherson and Sauder, 2013 ) or blend ( Binder, 2007 ), making organisations more enduring, sustainable and innovative ( Jay, 2013 ). Thus, the consequences of logic multiplicity vary and might depend on how these logics are manifested within the organisation ( Besharov and Smith, 2014 ). To better predict the outcome of external and internal demands, it is important to understand how institutional logics manifest within an organisation.

To understand how multiple logics manifest and their heterogeneity, two dimensions are essential: compatibility and centrality ( Besharov and Smith, 2014 ). Compatibility is the extent to which the presence of multiple logics within an organisation implies consistent organisational actions. Centrality describes the extent to which these logics are apparent in core features that are central to organisational functioning.

## Methods

An interview guide was designed based on the structure of the reimbursement programme. The interview guide was designed as an aide-memoire ( Burgess, 1991 ) to ensure all aspects are covered but still allowing for the informant to talk freely about the topics. To recruit informants for interviews, we used a purposive sampling approach ( Ruhl, 2004 ) in dialogue with the respective managers at the four clinics. We wanted the respondents to reflect the heterogeneity among staff. Thus, both clinically active and administrative staff were included from different professions to attain a more comprehensive perspective. Before commencing the fieldwork, we obtained ethical approval (2015/94-31) from the regional board of ethics in Linköping, as well as a signed consent to participate from each informant.

We conducted in total 41 semi-structured face-to-face interviews with staff members at all four of the accredited healthcare providers, at their respective spine surgery clinics. The interviews were carried out in two waves, from May 2015 to May 2016 and from June to September 2017. Seven informants were interviewed in both the first and the second wave, thus 34 unique informants were interviewed. Three interviews were conducted with two informants at the same time after a query from the informants. Two interviews were conducted over the telephone, both in the second wave, with informants who had already been interviewed face-to-face during the first wave. Each interview lasted between 20 and 60 min. All but one of the interviews were audio recorded and transcribed verbatim. To make the informants feel comfortable in the situation, each interview started with more general questions about the informant's profession and responsibilities ( Ruhl, 2004 ).

In our analysis, we divided the informants into five different groups based on occupation/function: spine surgeons ( *n* = 4), physiotherapists ( *n* = 4), nurses ( *n* = 8), managers ( *n* = 10) and administrators ( *n* = 8). In the managerial group, three out of six CEOs/operational managers were also working part time clinically as spine surgeons. Five out of the eight nurses also had administrative support functions and did not work fulltime clinically. The administrators group comprised bookkeepers and clinical invoicing assistants. The interviews were analysed using a thematic content analysis ( Ritchie *et al.* , 2013 ). We adopted an abductive approach that allows for interaction with previous and newly discovered knowledge, thus allowing for a combination of a deductive and inductive approach ( Alvesson, 2011 ).

The neo-institutional framework by Scott (2014) was used to structure the findings in order to challenge the mind to deepen the analysis and to connect the empirical findings to theory. An iterative process followed, where aspects within the respective pillars were identified. The identified aspects were compared between the groups to see if there were any differences and whether multiple institutional logics could be identified. Thereafter we analysed the relationship between the identified logics and how their centrality and compatibility was affected by the introduction of the new commissioning contract/VBRP.

The originators of the quotes used in “Findings” have been encrypted to ensure that individual informants cannot be identified. The informant will be denoted with the first letter of their group name followed by a number indicating the participant (i.e. Spine surgeon 1 = S1, Nurse 1 = N1, etc.)

## Findings

There was a variability in how the VBRP was perceived between and within the different actor groups within the healthcare-providing organisations. The identified institutional aspects among the actor groups are summarised in Table 1 .

### The regulative pillar

#### Management

It was stated in the commissioning contract that the patient should be assessed from an interdisciplinary perspective. However, the commissioning organisation did not impose any sanctions if this was disregarded. It should also be noted that some management groups were part of a larger organisation and had to manoeuvre additional demands external to the spine surgery clinic.

We have not been up and running for that long with this Patient Choice so … and the first year you mainly acclimatise and that sort of things. And then we went into our reorganisation in 2015, and then we didn’t have time with follow-ups. So it wasn’t until this autumn we made an effort to get it going. (M6)

Management received information on healthcare consumed by their patients after discharge. Among the costs for post-discharge care, physiotherapy was palpable along with the cost of treating infections. Thus, the bundled payment generated a new type of cost for management to take into consideration.

#### Spine surgeons

Spine surgeons were represented at a managerial level at all clinics, they were generally involved in all processes at the clinic. The spine surgeons were also represented in the process when the VBRP was developed. Hence, spine surgeons continuously received information about the new commissioning contract.

We get these bills from hospitals and you notice that, and from the county council, there is a long line of bills and if you check it then you see that there are lots of errors in it. (S4)

With the new commissioning contract, spine surgeons received information on what happened to their patients in post-discharge care, which had not been possible before. A side-effect was an increased administrative workload as they had to support accountants in the auditing of invoices on external care, because of IT-systems not being refined enough to distinguish related care from unrelated care.

#### Physiotherapists

Physiotherapists were not represented on a managerial level, nor in the development of the VBRP. Thus, physiotherapists had no immediate insight into what the new reimbursement programme would entail. Consequently, physiotherapists had to relate to the regulative framework depicted by the management, rather than the framework put forward in the commissioning contract.

We experience it as a small shortcoming that no one has looked at this agreement through the eyes of a physiotherapist. […] We have been busy working on the floor with patients. (P3)

However, physiotherapists experienced that physiotherapy gained more attention from both management and spine surgeons after the introduction of the VBRP, due to the invoices on external physiotherapy. Some physiotherapists were informed about the main features of the programme and were told to focus on how to improve physiotherapy, and how to make patients return to their own clinic for physiotherapy. Other physiotherapists were told not to refer patients to external physiotherapists as a consequence of the financial responsibility of post-discharge care.

#### Nurses

The amount of information nurses received about the new contract depended on the managerial strategy of the clinic. In general, nurses were less informed about the new regulative framework. Some nurses said that after the introduction of the VBRP, the reimbursement level was lower and in order to reach profitable volumes they had to receive more patients and increase efficiency.

Since we have to get more patients because the prices are not the same, we have to be more efficient. We have gone through every single task here, all the time and done everything as efficiently as possible. That's why we started with reception nurses […] to make it easier for the physicians so that they can operate more. (N7)

One strategy to increase efficiency was that nurses took over activities that were previously carried out by physicians in order to create more time for surgeons to assess patients and perform surgeries.

#### Accountant/clinical invoicing assistant

Accountants faced new stricter ways to register the combination of diagnosis and procedural code, which took some time to get used to. It was important to remind the spine surgeons to only use combinations that generated reimbursement.

The inflow of invoices for post-discharge care generated an additional process of auditing for accountants. Mainly because invoices for post-discharge care had to be audited manually due to the lack of data programmes that could properly assess the invoices and medical records. The accountants also had to involve spine surgeons since medical expertise was required to properly assess whether the external care was related to the spine surgery or not.

Yes, I have to spend more time auditing, so that has changed. Because there are more controls today, I have to sit and troubleshoot in a different way. (A1)

The performance-based payment was not possible to control because they did not know how the payment was calculated, hence they had to rely on the calculation made by the Region as accurate. Therefore, the performance-based payment was disregarded in the auditing, unless it had extreme positive or negative values.

### The normative pillar

#### Management

Some managers had long urged the region to abandon the Public Procurement Act and introduce patient choice within elective spine surgery. Consequently, some managers had been more involved when advocating for patient choice with value-based reimbursement, and thus more involved in the design process of the new commissioning contract. In general, managers thought VBRP seemed to be a good contract because of the involvement and the positive attitude of spine surgeons.

But I thought that the spine surgeons still felt that, they have developed a Patient Choice here that was thought through with a good introduction. (M7)

However, managers not directly involved in the VBRP, experienced that managers that had received first-hand information were better prepared for the new way of thinking and could therefore adapt their practice better. Managers said that an understanding of the contract and a good dialogue with the commissioning organisation was important for smoother implementation of the VBRP.

Very important, because changes are made, if we want to make a change, I must know whether I can get compensated for it or not. And then I must have an answer because it also takes time to work in various changes that ultimately create quality that enables us … So the dialogue is very important. (M5)

Management appreciated the use of a patient reported outcome measure in the performance-based payment. That it was about time the commissioning organisation followed-up on quality and not process measures that, according to them, said nothing about quality of care.

And what they control with, so called “you-shall-demands” that are really dumb and which they also don’t follow up on, and the demands are just formal and idiotic. (M1)

According to managers, the performance-based payment turned out to be too low in relation to the bundled payment to have any financial impact. Hence, the bundled payment imposed strong incentives to minimise post-discharge care. Among the costs for post-discharge care, the cost of treating infection and physiotherapy was palpable. Managers were prepared for this responsibility to varying degrees, and handled it in different ways. Some considered it as an opportunity to incorporate rehabilitation and post-discharge care as core values in their practice. Others focused on avoiding post-discharge care and rehabilitation as far as possible, since their main responsibility was spine surgery. Managers, who did not know how to handle this responsibility, slowly adapted the organisation to better facilitate physiotherapy by listening to suggestions by physiotherapists, but also by looking at solutions by other providers. Further, managers also differed in their encouragement of interdisciplinary assessment of patients. Some could see the benefits but thought it was too expensive, whereas others thought it was necessary and something that would pay off in the long run.

The patient experiences it as a good thing that the surgeon comes in and says that this is not a surgical case, listen to the physiotherapist. The physiotherapist gains more credence from the patient and the patient seems to absorb that information and try to listen and do something. So that's a WIN-WIN situation for everyone, it takes more resources from us but we think it's worth it at present. (M3)

An issue with interdisciplinary assessments was that patients often felt more reassured by receiving conservative treatment if the decision was delivered by a spine surgeon rather than a physiotherapist. The physiotherapist did not have the same legitimacy as the spine surgeon. Hence, when the surgeon and the physiotherapist had the discussion in front of the patient, the patient was further involved with the alternatives, but it was also a strategy to give legitimacy to the physiotherapist.

#### Spine surgeons

Spine surgeons exhibited a professional pride in the VBRP since their profession had been represented in the development process. Thus, spine surgeons had a positive attitude towards VBRP even though they did not fully understand it.

Yes, it was anchored and we had received a lot of information about it. Then, as I said, not everyone thought that they really understood how it would actually work out. (S1)

Spine surgeons experienced that the VBRP was aligned with professional values and appreciated that the bundled payment was combined with the performance-based payment. The use of Global Assessment (GA) was supported by the surgeons since it had been developed by spine surgeons and an established variable in the Swedish quality registry for spine surgery (Swespine). Initially, the performance-based payment caused them to discuss how the pain of the patient could be improved one year after surgery but soon the discussion was all about how to minimise the need of post-surgery care. They had to choose the surgical procedure with the best outcome over a year, with the least risk of complications and re-surgery. Spine surgeons expressed different perceptions regarding post-discharge care and interdisciplinary assessment of patients. Some emphasised that they had specialised in spine surgery, which does not include coordination of post-surgery care and physiotherapy.

They [physiotherapists] are not surgeons and this is a purely surgical business. (S3)

Others emphasised the importance of different perspectives when assessing the patient. Spine surgeons spent a great deal of time assessing referrals of patients with only a fraction of them being subjected to surgery. Thus, much time would be saved if other professions could help with non-surgery patients.

The spine surgeons also had strong opinions regarding management and they preferred if the manager of the clinic was a physician, preferably a spine surgeon.

Unfortunately this is how it has been, now it is my private opinion, but it is no use having a medical organisation run by nurses who are to be managers. It usually doesn’t work. Now she is a nurse, but in a completely different way, she includes us in the decision-making process and involves us. Because she has realised that it must be that way. (S4)

If the manager was not a physician, surgeons meant that he or she must involve the surgeons in the decision process. Surgeons expressed that other professions lack true understanding of spine surgery and show weakness with regard to the top management of the organisation.

#### Physiotherapists

In general, physiotherapists said that the discussions about physiotherapy had become more open now compared to in the beginning of the transition to the VBRP. Some meant that it was because of the high cost of physiotherapy in post-discharge care.

We have received several of those invoices from Region Stockholm, the costs of physiotherapy. So from a business point of view I think there has probably been a certain increased understanding of this, that physiotherapy exists as well and that this is something that needs to exist and to be included in the budget. (P3)

The experience of the VBRP among physiotherapists was heavily dependent on the strategy put forward by the management group. When physiotherapists could focus on how to improve the health of the patient, physiotherapists experienced the transition as smooth without any drastic changes. They also expressed the importance of the support from the spine surgeons in the transition to interdisciplinary care.

I think the surgeons have done a great job of demolishing these hierarchical blocks that I experience that major hospitals have had. There is always an open door, high ceilings, we can vent and discuss with them. (P2)

However, without support from spine surgeons and management in how to adapt to the new contract, physiotherapists experienced the transition as stressful. This was explained by lack of information and that no one had assessed the new reimbursement contract from the perspective of physiotherapy since physiotherapists had not been involved in the development process of the VBRP and lacked representation at a managerial level.

So I do not know the thoughts behind the whole contract, but I am curious about it. (P4)

In the assessment of indications for surgery, physiotherapists meant that they could better assess the physiotherapy the patient had undergone previously. Physiotherapists emphasised the quality aspects of physiotherapy and the importance of communication between surgeons and physiotherapists.

It's difficult because we, our efforts, do not cause the patient to live or die, so to speak. So that there are no such efforts that are vital in that way, but on the other hand there are quality aspects related to our license as a physiotherapist. (P3)

However, physiotherapists experienced that the quality aspect often got neglected due to the association between physiotherapy and high costs of post-discharge care. The focus was on how to minimise physiotherapy rather than providing high quality physiotherapy. Physiotherapists experienced conflicting demands when spine surgeons and management neglected the quality aspects of physiotherapy in spine surgery care.

#### Nurses

Nurses did not experience the introduction of the VBRP as much different from ordinary quality improvement work.

I would say that since I started here, it feels like it is under constant change all the time. We change things all the time and adapt the business to new circumstances. So I think there wasn’t a huge change for us when the new contract was introduced. (N2)

There were also nurses that experienced that after the introduction of the VBRP, they had more time for reflection and better communication with the surgeons. Which they said was important to provide high quality care. However, other nurses experienced that they had less time with each patient due to financial cuts because of the VBRP. These nurses meant that so far, it had led to a more efficient practice and for the better, albeit challenging. However, if they had to continue to make cuts they worried it would negatively affect the quality of care.

We have provided a very high level of nursing and had the space to give that little extra. Not that we don’t give the patient adequate care, because we do. But that little extra, sometimes you don’t have time for it anymore, unfortunately. […] That you take some extra walks, you go with them to the dining room, that you sit down and talk for a longer time. These are small things, it does not have any effect but it affects the patient mentally, so to speak. (N7)

Hence, quality aspects of nursing was neglected to better facilitate the ability to perform surgery after the introduction of the VBRP.

#### Accountant/clinical invoicing assistant

The administrative staff had a rather challenging process when transitioning to the new system. Accountants faced rather explicit problems in the beginning, such as incompatible IT-systems or procedures that were not covered by the contract. The fact that spine surgeons had been active in the design of the VBRP and their positive attitude towards it, lent credence to the introduction of the VBRP for administrative staff, despite the increased administrative workload.

We lack information, what to actually do and who to turn to and who can answer questions? Our contact persons at Region Stockholm do not really know either, it's too big. (A6)

They also experienced a lack of communication, especially regarding technical issues. It took a long time before their questions were answered, if answered at all. However, the initial confusion subsided during the initial two years and administrative staff felt more secure in their responsibilities.

There were completely different requirements when registering, so there were a lot of questions like “is this right?” and so on. But it has actually settled down now. (A2)

Thus, in the second wave of interviews, informants said that they had learnt more about the regulative framework. They also thought that the commissioning organisation had learnt more about the framework since they responded faster to questions.

### The culture-cognitive pillar

#### Management

Management had a positive attitude to the holistic perspective on spine surgery that the VBRP entailed. However, the extent of the bundled payment had not been fully understood among management groups that were not directly involved in the design process. All management groups did not fully grasp the new contract at the time of the introduction of the VBRP. Without instant insight in the design of the reimbursement programme, management groups described the VBRP as a completely new world they did not know how to adapt to.

Then a person, a representative, was appointed from one of the other [clinics] and that meant that we did not have quite the same insight, even though it was a person who certainly reported to us and asked questions “What do you think about this?”. But we were never involved in the work itself, so when these regulations came, it was like a whole new world that opened up, with everything that involved. (M4)

Managers that were not fully prepared (since they had not participated in the development process) said that they had no shared logics of action and therefore struggled in how to translate the contract into practice. They had not understood how extensive the new commissioning contract was. However, management at all clinics was strongly influenced by the positive opinion among spine surgeons.

So I think there is a fundamental positive attitude even if people don’t really understand what it is. (M1)

After the introduction of the VBRP, the first years had mainly been devoted to understand the new contract. Focus had been on adjusting the administrative parts to make sure they would receive reimbursement for their services. Hence, not much thought had been put into how to develop their practice to attain more integrated care with a more holistic perspective.

In recent years, or during the time I've been here, the biggest change is that we have learned more about the contract. (M5)

The cost responsibility for post-discharge care made physiotherapy a challenge, since it required another way of thinking of the care chain. Thus, management could choose to offer patients physiotherapy “in house” or by referring them to contracted physiotherapists. There was no shared logics of how to take on the greater responsibility for post-discharge care in general, and physiotherapy in particular.

Due to the lack of financial impact of the performance-based payment and no logics of action on how to improve the pain one year after surgery, the dominating logic among managers was to minimise cost instead of maximising quality. This made it more important to focus on how to minimise costs of post-discharge care and increase the surgery volume.

The driving economic forces are to reduce complications, to look at the physiotherapy, to look at the parts rather than increase the performance, it's not that damn easy to just increase performance. (M1)

Hence, management struggled with how to transition to a holistic and integrated care-chain with interdisciplinary assessment of patients.

#### Spine surgeons

Surgeons experienced that their way of thinking about spine surgery was challenged by the introduction of the VBRP that entailed a unique opportunity to receive information about post-discharge care. Spine surgeons had no mental framework on how to manage this increased responsibility for post-discharge care and their approach depended on whether they considered their clinic to be “a purely surgical business” (S3) or not. Spine surgeons also said that there was not enough time for research and education.

It has been a negative consequence, in order to increase time for surgery and reception and yes our availability, these areas have suffered, unfortunately. Research is less prioritised now, it has ended up a little further down the list which isn’t good. (S4)

Other surgeons meant that research was crucial to develop spine surgery and the VBRP had not affected their ability conduct research. The indications for surgery are at times rather vague and the evidence for when surgery is to be preferred compared to conservative treatment is still unclear. Thus, it is difficult to know who will benefit from surgery and who will not. With the increased cost-responsibility for post discharge care, there is a risk of cherry-picking. However, due to professional values, competition and low reimbursement levels, spine surgeons felt that they could not be picky. More research is needed on how to improve the surgical assessment but also on how to improve the surgical result and minimise post-discharge care.

#### Physiotherapists

Physiotherapists expressed that the VBRP had put a focus on physiotherapy in a way that would not have happened otherwise, now managers and surgeons paid more attention to, and interest in, physiotherapy.

Yes, the thinking in any case, that it would be good to offer it [physiotherapy] and why it would be good and things like that. And that you try to assess patients from an inter-professional perspective and value that higher I definitely think, but there is not enough time for that sort of collaboration right now. (P3)

Physiotherapists have pride in their work and are confident that physiotherapy can be a crucial factor in whether a surgery is successful or not. Physiotherapy provides continuous contact with the patient during a longer time period as compared to the surgical procedure. They found it challenging to find new ways of working and taking professional responsibility if management intervened, making them do things in a different way.

I feel really sad about it and I think it's pretty difficult to work that way. It would be nice to have an understanding of why I say it … I do as I'm told but that doesn’t always feel that good. (P4)

It was difficult for physiotherapists to take full responsibility for quality aspects of physiotherapy at a spine surgery clinic unless they had full support from management and spine surgeons.

#### Nurses

There were differences in how nurses talked about their role and responsibilities. All nurses said that their role was to care about and comfort the patient before and after the surgery, but also to coordinate care. Some nurses emphasised nursing as a discipline, how important it was to stay á jour with the latest research and find solutions to problems they experienced.

It is our responsibility to stay up to date. We are responsible for the materials we use and other developments within healthcare. (N3)

Thus, contributing to the team at the clinic with their expertise within nursing/caring sciences. Others emphasised the importance of facilitating surgeons even if it meant that quality aspects of nursing were neglected in order to become more efficient. However, the care they provided was still adequate but not with as high quality as preferred since “it does not have any effect but it affects the patient mentally” (N7), thus subordinated spine surgery.

#### Accountant/clinical invoicing assistant

Accountants were in general positive to the VBRP, but the performance-based payment was considered too complex due to lack of transparency and lack of financial impact.

Yes, it is clear that the new contract entails complexity, which is basically good, it is good that it is quality-based, etc., it is very, very good. But if we just look from the financial side, it has of course become incredibly more complicated. (A1)

Thus, in order to operate within complex reimbursement programmes the technical infrastructure must be able to handle the interlinking of data in a smoother way. Otherwise healthcare providers cannot follow-up on their performance properly, neither medically nor financially.

## The centrality and compatibility of institutional logics

Our analysis shows that the spine surgery logic was dominant (“a purely surgical business” S3) and aligned with managerial and accounting logics; other logics existed in the periphery. Spine surgeons, managers and administrators emphasized clinical aspects in combination with financial aspects. The logic of physiotherapists and nurses were peripheral with a more supporting function, since their “efforts do not cause the patient to live or die” (P3). Before the VBRP, The peripheral logics seemed to be assimilated with the core logic. The introduction of the VBRP reinforced a more holistic perspective and physiotherapy reached a more central position due to the high costs for post-discharge care. Thus, the centrality of physiotherapy increased. However, this did not automatically mean a high compatibility. The centrality and compatibility of the identified logics are described in Table 2 .

The compatibility decreased if managers minimised physiotherapy to decrease costs and thus neglected the quality aspects within the physiotherapeutic profession. This caused moral dilemmas among physiotherapists and paved the way for growing discontent since their logic was neglected. If information about the contract was buffered through management, and spine surgery was dominating, this only caused a modest conflict. However, it caused ethical stress among physiotherapists. If information was not buffered, physiotherapists realised the increased centrality of physiotherapy, but also that they would have to fight for their professional values. Hence, physiotherapists contested spine surgeons' dominating logic and the compatibility decreased initially. However, with time the compatibility increased when the different professions built new mental frameworks on how to cooperate. Thus, moving from being a dominated organisation, to a contested one and after some years approaching being an aligned organisation.

Managers and spine surgeons advocated to increase efficiency by letting nurses perform tasks that were previously performed by surgeons. Hence, nurses were not able to provide the same care as before the VBRP since they had less time with each patient. This did not, however, cause any estrangement since the nurses experienced the work of surgeons as superior to their work as nurses. As one nurse reflected about it as “These are small things, it does not have any effect but it affects the patient mentally” (N7). Other nurses experienced that the VBRP enabled them to think differently about the care chain in a more holistic way. They also expressed the importance of being encouraged by managers and spine surgeons to focus on nursing and to find new solutions within their field of expertise.

After the introduction of the VBRP, two strategies among managers and spine surgeons became more distinct; encouraging peripheral logics to facilitate spine surgeons (the dominating logic) or encouraging higher centrality of peripheral logics to facilitate a holistic healthcare perspective. The centrality of previously peripheral logics increased when managers and spine surgeons acknowledged the importance of other logics in spine surgery care. Thus, the centrality and compatibility of logics of physiotherapists and nurses was dependent on how managers and spine surgeons considered the mission/purpose of the spine surgery clinic.

## Discussion

In our analysis it became apparent that not all occupational groups experienced any of the intended incentives within the reimbursement programme. We found that external demands affected existing institutional logics within organisations differently, depending on the managerial and spine surgeon logic. In our material we found evidence of how the centrality and compatibility of logics changed after the introduction of the VBRP. This study contributes to theoretical and empirical explanations of why reimbursement programmes do not always have the intended effects.

By using Scott's framework of institutional pillars we were able to identify different aspects of institutions. From a regulative perspective it was evident that not everyone was informed about the regulative changes, since information was “buffered” through management. This has previously been shown to affect whether healthcare providers adapt to regulative changes ( Ellegard and Glenngard, 2019 ). Further, it was also clear that management had other demands to take into account, which was evident when quality aspects of physiotherapy were neglected. This can be seen as a side effect of incentives that were too strong to minimise post-discharge care, which rather put more emphasis on the surgical procedure than other activities which could enhance the result.

From a normative perspective our study shows that spine surgeons asserted a strong influence on other logics. Multiple institutional logics have traditionally been handled through a hierarchy where the medical logics of physicians have dominated other logics. However, transitioning to more holistic healthcare allows other logics to reach higher centrality. In most countries, healthcare is organised based on the specialty of the physicians. Our findings show that this structure may hamper the transition to more integrated care. To transition from a physician dominated logic to an interdisciplinary logic may be difficult if the organisation is a “hostage to [its] own history” ( Selznick, 1992 ). When the spine surgery logic was dominating the healthcare providing organisation, it was difficult for other healthcare professionals to stand up for their professional values if they generated additional costs. Further, spine surgeons had strong opinions regarding the management of the clinic, especially that the clinical director should preferably be a spine surgeon. However, to attain a holistic perspective of healthcare it is important to have an understanding of different professions and their logics.

Our findings show that the different stakeholders within a healthcare providing organisation had different institutional logics. The logic of management and spine surgeons was very similar, except that managerial representatives more often expressed a more holistic perspective of the role of the spine surgery clinic. Management groups that were less prepared for the transition to the VBRP, were also more resistant to the new ideas and how extensive the changes of their practice had to be, thus they were more reluctant to change. We also found evidence of “decoupling” as some managers accepted a higher cost for post-discharge care rather than adjusting their practice. The development of more holistic care requires shared understanding of the process amongst co-workers. Establishing understanding requires leadership skills such as communication with, and motivation of, involved staff ( Nilsson and Sandoff, 2015 ). It is also important to acknowledge the importance of managers who can dampen and modify external demands ( Ellegard and Glenngard, 2019 ). Further, our study showed that professional groups that lacked representation in the design of the programme, considered that as a problem, since each professional category can contribute with different perspectives in the care chain.

Our study was set in private organisations affected by a specific policy reform, entailing patient choice and a value-based reimbursement programme. Our results may be limited in terms of their generalisability to other specialties or other healthcare systems. Yet, the focus on the different professional groups, such as physicians, managers and physiotherapists may be important knowledge in any healthcare system or specialty, since it highlights the different institutional settings for each professional group.

Further, as shown by our study, combining institutional pillars ( Scott, 2014 ) and institutional logics ( Thornton *et al.* , 2012 ) can enhance our knowledge on how and why external demands are institutionalised or not.

## Summary

This paper contributes empirically to the theory of how external demands are institutionalised within an organisation consisting of multiple institutional logics. The introduction of the new contract had a destabilising effect when the dominating logic did not support higher centrality of peripheral logics. The centrality of physiotherapy increased through both external and internal pressure.

## Figures and Tables

**Table 1:** Institutional aspects brought up by the five actor groups based on the regulative, normative and culture-cognitive pillars

	Regulative	Normative	Culture-cognitive
Managers	Increased financial responsibility generated information about post-discharge care	Strong incentives to minimise post-discharge care. How to involve other professions differed	The VBRP imposed a holistic perspective on healthcare, which managers embraced differently
	Management may have additional external demands to take into account, affecting the response to the new commissioning contract	Highly influenced by spine surgeons' perception	Time to comprehend and understand the new contract is important in finding strategies when adapting the business
		Crucial with a good dialogue with the commissioning organisation	
Spine surgeons	Increased financial responsibility generated information about post-discharge care	The significance of involving other healthcare professionals in spine surgery was debated and whether post-discharge care should be their responsibility	Broaden their perspective to see the entire care chain instead of only the surgical procedure
Physiotherapists	Information about the VBRP was buffered through management and spine surgeons	Opposed to their morals when physiotherapy was seen as a cost that should be minimised rather than a quality aspect	Difficult for physiotherapist to find their way of contributing when the focus was on how to facilitate spine surgery
Nurses	Less informed about the new regulative framework	It was unfortunate that quality aspects within nursing had to suffer in order to better facilitate surgeons	Nurses were responsible for providing adequate care from a nursing perspective but functioned also as a facilitator for spine surgery. The introduction of the VBRP refined the role of nurses to focus more on one of the two aspects
	Some received new tasks to ease the workload of surgeons to increase efficiency	Taking responsibility for quality aspects within nursing requires air in the system to allow reflection and quality assessment	
Administrators	The VBRP entailed more manual auditing when assessing invoices and medical records	Increased workload that also made administrators dependent on surgeons. However, the positive attitude from spine surgeons made the workload acceptable	Impossible to do their work without supporting infrastructure

**Table 2:** Centrality and compatibility of identified institutional logics among actor groups within healthcare providers in elective spine surgery

Group	Centrality	Compatibility
Managers	*High*	*High and low*
	Managers were an essential link to receive reimbursement and facilitate provision of healthcare	Managers had strong impact on other actor groups but were highly influenced by spine surgeons. Neglecting quality aspects of peripheral logics could cause contestation and estrangement
Spine surgeons	*High*	*High and low*
	Spine surgeons had the knowledge of how to perform the surgeries that generated reimbursement. Also crucial to assess invoices after the introduction of the VBRP	The spine surgeon logic had strong influence on other logics within the organisation. Following VBRP, the dominance of the spine surgery logic was challenged by other logics. If spine surgeons were unwilling to allow higher centrality for other logics, the compatibility decreased and the balance between logics was estranged
Physiotherapists	*High and low*	*High and low*
	Peripheral in facilitating spine surgery. Central to improve back problems and minimising cost of post-discharge care	If physiotherapists experienced support from managers and/or spine surgeons, the logics attained high compatibility. If physiotherapists experienced that quality aspects were neglected, the compatibility was low
Nurses	*Low*	*High*
	Nurses cared for patients before and after surgery. Nurses facilitated surgeons by taking over tasks previously performed by surgeons	Having a supportive function in facilitating spine surgeons and contributing with a nursing perspective. Following VBRP, the role of nurses was refined with a more clear focus on one of these aspects
Administrators	*High*	*High*
	Crucial to receive reimbursement by sending correct information to the commissioning organisation and assessing invoices	Having a supportive function in facilitating provision of healthcare
